# Strategies to Improve Participation of Older Adults in Cancer Research

**DOI:** 10.3390/jcm9051571

**Published:** 2020-05-22

**Authors:** Jennifer Liu, Eutiquio Gutierrez, Abhay Tiwari, Simran Padam, Daneng Li, William Dale, Sumanta K. Pal, Daphne Stewart, Shanmugga Subbiah, Linda D. Bosserman, Cary Presant, Tanyanika Phillips, Kelly Yap, Addie Hill, Geetika Bhatt, Christina Yeon, Mary Cianfrocca, Yuan Yuan, Joanne Mortimer, Mina S. Sedrak

**Affiliations:** 1 Department of Medical Oncology and Therapeutics Research, City of Hope, Duarte, CA 91010, USA; jennliu@coh.org (J.L.); atiwari@coh.org (A.T.); spadam@coh.org (S.P.); danli@coh.org (D.L.); SPal@coh.org (S.K.P.); dapstewart@coh.org (D.S.); ssubbiah@coh.org (S.S.); lbosserman@coh.org (L.D.B.); cpresant@coh.org (C.P.); taphillips@coh.org (T.P.); keyap@coh.org (K.Y.); ahill@coh.org (A.H.); gbhatt@coh.org (G.B.); cyeon@coh.org (C.Y.); mcianfrocca@coh.org (M.C.); yuyuan@coh.org (Y.Y.); jmortimer@coh.org (J.M.); 2 Department of Internal Medicine, Harbor-UCLA Medical Center, Los Angeles, CA 90502, USA; EGutierrez3@dhs.lacounty.gov; 3 Department of Supportive Care Medicine, City of Hope, Duarte, CA 91010, USA; wdale@coh.org

**Keywords:** geriatric oncology, older adults, cancer clinical trials, recruitment, community, team science

## Abstract

Cancer is a disease associated with aging. As the US population ages, the number of older adults with cancer is projected to dramatically increase. Despite this, older adults remain vastly underrepresented in research that sets the standards for cancer treatments and, consequently, clinicians struggle with how to interpret data from clinical trials and apply them to older adults in practice. A combination of system, clinician, and patient barriers bar opportunities for trial participation for many older patients, and strategies are needed to address these barriers at multiple fronts, five of which are offered here. This review highlights the need to (1) broaden eligibility criteria, (2) measure relevant end points, (3) expand standard trial designs, (4) increase resources (e.g., institutional support, interdisciplinary care, and telehealth), and (5) develop targeted interventions (e.g., behavioral interventions to promote patient enrollment). Implementing these solutions requires a substantial investment in engaging and collaborating with community-based practices, where the majority of older patients with cancer receive their care. Multifaceted strategies are needed to ensure that older patients with cancer, across diverse healthcare settings, receive the highest-quality, evidence-based care.

## 1. Introduction

**Figure 1:**
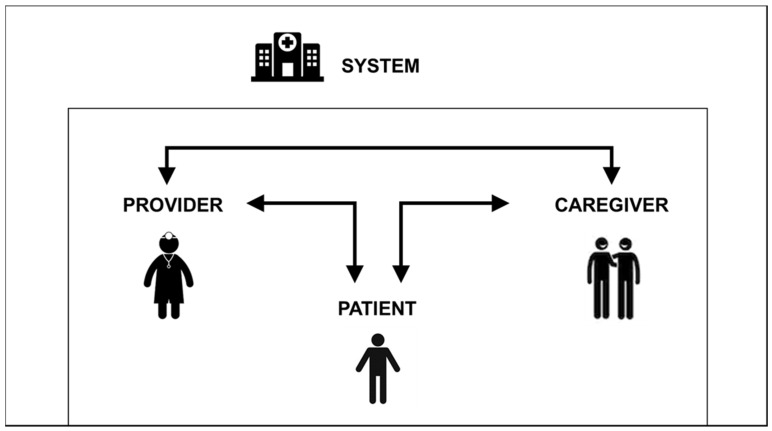
The barriers to older adult participation in cancer clinical trials are multifaceted and interrelated, with barriers existing at the system, clinician, patient, and caregiver levels. System-related barriers include trial design, overly stringent eligibility criteria, and lack of infrastructure support, appropriate and representative trials, and funding. Clinician-related barriers include concerns for side effects or toxicities, patient age, comorbid conditions, personal bias, costs to clinicians, and lack of time, support and staff, awareness of trials, and engagement amongst clinicians. Patient-related barriers include attitudes towards clinical trials, knowledge, side effects/toxicities, burden, and financial limitations. Caregiver-related barriers include caregiver burden and preference against participation.

Aging is a major risk factor for cancer, with 28% of cancers in the US diagnosed in adults aged 65–74, 18% in adults aged 75–84, and 8% in adults aged 85 and older [1]. Globally, the US has the third largest number of older adults [2], and as the US population ages, the number of older adults with cancer is increasing and will make up a growing share of the oncology population [3,4,5]. Despite this, older adults remain vastly underrepresented in the research that sets the standards for cancer treatments [6,7]. Consequently, most of what is known about cancer therapeutics is based on clinical trials conducted in younger and healthier patients [8,9,10]. Furthermore, despite a plethora of literature documenting barriers to the accrual of older adults to cancer clinical trials (Figure 1), there are few evidence-based strategies to mitigate these barriers [11,12]. A recent systematic review revealed that among 8691 studies screened, only 12 relevant observational studies examined barriers hindering the participation of older adults in cancer clinical trials, and one (negative) randomized controlled trial evaluated an intervention to increase the enrollment of older adults in trials [8]. Moreover, few studies have focused on understanding the complex and multifactorial influences affecting the clinical trial participation of older patients with cancer in the community, where the majority of this population is often treated [13,14].

Recognizing this problem, numerous organizations, including the Institute of Medicine (IOM), have cited the growing population of older adults with cancer and highlighted the need to generate high-quality, evidence-based data for the treatment of older adults [6,15,16,17,18]. The primary aim of this review is to lay out a multipronged approach that addresses barriers to the participation of older adults in cancer clinical trials. A secondary aim is to highlight how a collaborative clinical research network of academic and community-based oncology practices can facilitate the inclusion of older adults in cancer research.

## 2. Make Eligibility Criteria More Inclusive and Less Restrictive

Restrictive eligibility criteria often exclude a large proportion of patients from participating in clinical trials—a loss which sacrifices the generalizability of results to the overall patient population. Older adults, in particular, are often not offered the opportunity to participate in trials due to concerns for multiple comorbidities, organ dysfunction, treatment toxicity, and/or frailty [8,19,20,21,22]. Numerous trials explicitly restrict inclusion to patients with an Eastern Cooperative Oncology Group (ECOG) performance status of 0 to 1 or a Karnofsky performance score (KPS) of ≥70 [23,24]. As a result, trial data and findings are often derived from younger patients who are more fit and without organ dysfunction and comorbidities, as commonly seen in older adults [23].

A variety of efforts are underway to broaden the eligibility criteria to make trials more relevant to patients of all ages, including initiatives by the National Institutes of Health (“Inclusion Across the Lifespan policy”), American Society of Clinical Oncology (ASCO), and the Food and Drug Administration (FDA) [16,25,26]. Sponsors and investigators are encouraged to work together to follow these policy recommendations aimed at making the eligibility criteria less restrictive and more inclusive of demographically and clinically diverse patients, representative of the populations seen in community-based oncology settings. Investigators should review the eligibility criteria closely and forgo exclusion on the basis of lab values (e.g., creatinine), performance status, comorbid conditions, or second malignancy when designing clinical trials. Instead, a patient’s functional or biological age should be taken into consideration, which may be a better indicator of how a patient will tolerate a certain treatment regimen.

Additionally, investigators should work closely with community clinicians and other stakeholders (i.e., patient advocates) to understand the patient populations seen in community-based practices and design eligibility criteria that are more inclusive, thus allowing trial results to be applicable to a broader patient population. Inclusive clinical trial systems within a collaborative research network ought to consider patients as they are, rather than as they should be. Trial eligibility should be evaluated and revised to ensure that investigators are broadening study access to new, successful cancer treatment regimens for older adults with cancer across diverse healthcare delivery settings.

## 3. Capture Relevant End Points that Matter to Older Patients

Sponsors and investigators should capture relevant end points that are important to the older patient, including measures of tolerability as well as clinical and biological aging consequences of cancer and its treatment [17,27,28,29].

Studies have shown that most older adults do not want to compromise their quality of life or function for survival benefits [30]. Hence, trials designed to collect information on this population must include patient-reported measures that are relevant to the patient’s experience and move beyond clinician-reported toxicity. Therefore, data are needed to understand the immediate (short-term) and longitudinal (long-term) impact of cancer and cancer therapy on patient health and quality of life [29,31]. Given that each individual is unique and can respond differently to therapy, incorporating assessments that focus on patient-reported outcomes may allow investigators to better describe a patient’s tolerability beyond the traditional numerical grades (NCI CTCAE) [32,33,34], and the use of mixed-methods approaches (e.g., surveys, interviews, focus groups) may allow investigators to better understand a patient’s experience and priorities for treatment. Furthermore, measuring the frequency of toxicities over time can help investigators to characterize the longitudinal impact of treatment and better determine the relevance to the older patient population. This information can inform new ways treatment approaches and optimal strategies to reduce toxicities, avoid early treatment discontinuation, and achieve an effective dose intensity.

In addition to capturing measures of quality of life and tolerability, clinical measures of aging should also be considered as end points in cancer trials of older adults. This is important because aging is a heterogeneous process. While certain declines in organ function are universal as the human body ages, the consequences of this decline on everyday function proceed at a unique pace in each individual [35,36]. Therefore, chronologic age tells us relatively little about a specific individual [37]. A more detailed evaluation of an older patient is needed to capture factors that more effectively predict morbidity and mortality. A geriatric assessment (GA) may serve this purpose. The GA includes an evaluation of functional status, co-morbid medical conditions, cognitive function, nutritional status, social support, and psychological state, as well as a review of medications [38]. Additionally, geriatric screening tools (i.e., G8, VES-13), instruments that assess life expectancy (i.e., ePrognosis), and predictors for risks of chemotherapy toxicity (i.e., CARG or CRASH Score) can be utilized in trials to better describe or risk-stratify this population [39,40,41]. The ASCO guidelines now recommend the use of these assessments in the evaluation and management of older patients with cancer, and several NCI-sponsored cooperative groups have demonstrated the feasibility of using these measures in clinical trials [40].

Biological measures of aging are also important to consider when designing studies for older patients with cancer. These measures may provide insights on mechanisms behind aging-related clinical consequences due to cancer treatment, such as the biological drivers of functional and cognitive decline. Several studies have hypothesized that cancer and cancer treatment may accentuate or accelerate the rate of aging, leading to a decrease in multisystem reserve and increased risk for cardiomyopathy, secondary malignant neoplasms, frailty, muscular weakness, and neurocognitive issues [42]. Biological processes including stem cell exhaustion, cellular senescence, telomere attrition, increased free radical production, and epigenetic modifications have all been shown to play a role in accelerated aging due to cancer therapies [42,43]. Understanding the hallmarks of aging and the implications of cancer therapies associated with accelerated aging may provide insights into the short- and long-term effects of certain therapies, and further research in this area is needed. Knowledge of the underlying mechanisms to accelerated aging could ultimately help us to identify new strategies for targeting these processes in order to ameliorate accelerated aging and improve the health and well-being of this growing population.

## 4. Optimize Trial Designs for a Special Population

Inclusion of older patients may, in some cases, impose the need for larger sample sizes, which is not always feasible or fundable. Sponsors and investigators should consider high-yield approaches to collect data on older patients to efficiently facilitate the conduct of larger-scale trials at a reasonable cost. For example, pragmatic or innovative trials (e.g., adaptive, extended, embedded, N-of-1 studies) designed specifically for older patients may be used to fill knowledge gaps and improve the evidence base guiding the treatment of older adults [29,44].

There are several innovative trial designs that may be leveraged when considering a special population such as older adults with cancer (Table 1 adapted from prior literature [16,39]). For example, adaptive trials can be leveraged to allow for modifications to be made as the study proceeds [45,46]. Based on interim data analysis, the underperforming treatment arm may be eliminated to allow for a larger proportion of participants to be assigned to the more effective treatment arm. This is ideal for older adults because it reduces the number of participants in the treatment group that is performing poorly [16,39,47]. While adapted trial designs can be applicable to both exploratory and confirmatory studies, prospective cohort studies can be used to generate data on current standard-of-care treatments in older patients and provide insight into patterns of care and decision making [16,39].

Extended trial design can also be used in cases where the results of a trial have been reported, but there was an insufficient number of older adults enrolled to draw conclusions. A cohort of older adults can be added to the superior treatment arm to fill knowledge gaps and obtain data on the older population [16]. Embedded studies, also known as correlative or ancillary studies, can also be used to include additional measures of interest that are specific to older adults (i.e., toxicity, GA domains) within the infrastructure of a parent study [39]. Lastly, an N-of-1 or single-subject trial presents a feasible and innovative approach to better understanding how to care for older adults by following one patient over time, to examine aging-related consequences that occur throughout the cancer continuum [48].

**Figure 2:**
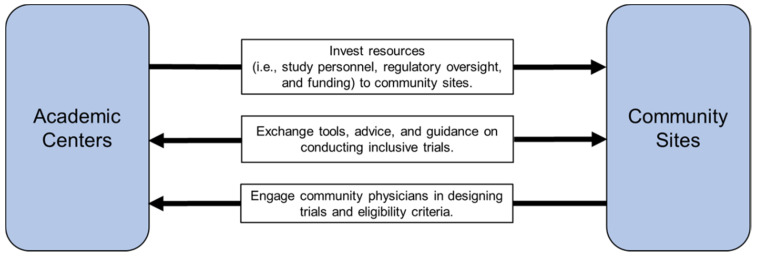
Strategies to connect academic centers and community-based practices. Ensuring collaboration between academic centers and community sites is essential in improving the participation of older adults in cancer research. First, academic centers should invest resources such as study personnel, regulatory oversight, and funding to community sites in order to help create trials and participation where most patients receive treatment. Second, both community sites and academic centers need to exchange tools, advice, and guidance to help increase the quality of trials being done and make sure that the needs of older adults are met. Lastly, community sites must have their clinicians participate in the designing of trials and eligibility criteria so that trials better fit the present patient population.

When designing clinical trials appropriate for the older patient population, incorporating older adults into the study design phase, such as through the utilization of community-based participatory research methodology, may facilitate their participation in clinical trials [49,50]. Moreover, it is important for sponsors and investigators to engage all key stakeholders, including patient advocates and community-based clinicians, in the process of trial design in order to understand the types of patients seen in community practices (Figure 2). These discussions can inform protocol design and ensure participants are more representative of the patient populations beyond the academic setting. Furthermore, these collaborations can foster improved accrual, conduct, and dissemination of the research, thus ultimately increasing generalizability and clinical relevance of the trial findings.

## 5. Increase Institutional Support, Interdisciplinary Care, and Telehealth Use

Structural barriers at the system or institutional level dominate trial decision-making, and successful participation in research requires substantial institutional guidance, resources, and infrastructure [51]. There are many barriers to the conduct and support of a clinical trial research program. Studies have shown that community-based practices often struggle with understanding the value of trials, covering the costs of supporting a research program, meeting program requirements, managing the clinic workflow changes as they pertain to clinician involvement, and sustaining hospital leadership support, among other barriers [13,52,53]. In order to overcome these barriers to entry, partnerships with larger practices or academic centers with existing infrastructure can facilitate the conduct of clinical trials at community practices. Additionally, leadership is needed at the institutional level to ensure that the mission, interests, and workflow of community sites are aligned with the academic medical centers in order to cover the ever-increasing requirements of implementing and maintaining a clinical research program, especially one that aims to increase the representation of older adults with cancer (Figure 2) [54].

In addition to institutional leadership, accessibility to specialized geriatric care may be an important facilitator in the recruitment and retention of older adults in cancer clinical trials. Clinical programs that meld geriatric and oncology communities to meet the complex needs of older adults may facilitate the improved management of older adults [17,30,39]. City of Hope, for example, has jointly launched two interdisciplinary clinical programs: (1) the Specialized Oncology Care & Research in the Elderly (SOCARE) clinic and (2) the Aging Wellness Clinic. Similar models have also been established at the Memorial Sloan Kettering Cancer Center, Thomas Jefferson University, and the University of Rochester, among others [55,56,57]. These specialized programs offer interdisciplinary, individualized, and integrated treatment for older patients and survivors with cancer. However, these programs require resources and institutional support, and further research is warranted to understand how these care models lead to improved patient outcomes and participation in clinical trials.

Furthermore, the integration of technology in the form of virtual visits and telehealth encounters may facilitate the enrollment of older adults in cancer research. There are two potential strategies for this. First, technology may be leveraged to reduce the burden of the patient and caregiver—a known barrier to older participation in research [8,17,39]. Studies have shown that telehealth use is associated with high patient satisfaction, improved access to care, and reduced health disparities among older adults [58,59,60,61,62]. Challenges to telehealth use among the older patient population must be taken into consideration, including practical barriers to adoption, anxiety using technology, and financial burden, among other factors that promote the digital divide [53]. To address some of these barriers, simple and intuitive graphical interfaces, the availability of technical support, and the provision of telehealth services at no cost may reduce the barriers to entry and improve adoption of telehealth in this population [63]. However, further efforts are needed to better understand the limits and benefits of telehealth in geriatric oncology. Moreover, the COVID-19 pandemic has led to the rapid implementation of telehealth services for both standard care and clinical research [64,65]. This swift paradigm shift in how patients receive their care provides a unique opportunity to gain insight on how telehealth can further be used to improve patient participation in cancer clinical trials—particularly in older adults. However, the feasibility, adoption, and sustainability of telehealth in clinical trials is limited, and further research is needed [66].

Second, telehealth can be a key player in providing specialized geriatric care to sites that may not have access to multidisciplinary programs with geriatric expertise. For example, City of Hope has an ongoing pilot study to evaluate the feasibility of delivering a GA-driven intervention at a community-affiliated site using telehealth. In this way, telehealth may help fill the gap in care for older patients who otherwise would not have access to multidisciplinary, specialized geriatric-based care [67]. Whether this improves clinical trial accrual warrants further investigation.

## 6. Leverage Principles of Behavioral Economics

There is a growing interest in the general medical literature around the use of behavioral economics to shape clinician and patient behavior, with some even suggesting the use of “nudges” to facilitate patient enrollment in research [68]. A nudge is defined as a change in the way choices are presented or information is framed that alters a person’s behavior in a predictable way without restricting choice [68,69]. If implemented properly, nudges are transparent, easy to opt out of if needed, and aligned with the best interest or welfare of the person being nudged. It is a principle of behavioral economics that leverages the fact that our decisions and behaviors are heavily influenced by the environment in which they occur, and interventions can be systematically developed to influence how individuals behave (e.g., weight loss, exercise, and statin utilization) [70,71,72,73,74].

Nudges may also be a novel way to facilitate patient participation in cancer clinical trials, especially among highly underrepresented groups such as older adults [75]. For example, surveys that assess patients’ interest in participating in clinical research can help identify eligible patients who are likely to participate and provide insight into the types of trials that are desirable. Using these interest surveys, clinicians can identify patients who may be more inclined to participate in studies when presented the opportunity to do so due to their prior expressed interest (i.e., foot-in-the-door technique) [75]. Another nudging strategy that can be utilized to encourage the enrollment of older adults in cancer trials is the personalization of trials [75]. Explaining why a patient was specifically chosen to participate, how they may personally benefit, and how their participation will contribute to the scientific community and future patients may improve patient enrollment. Furthermore, nudges can be directed towards providers. Clinicians, for example, can be nudged by incorporating the trial information in the clinical pathway of electronic health records [69]. Including the trial information in electronic health records may improve clinician awareness of potentially beneficial trials regarding their patients and help facilitate enrollment of older adults.

Evidence on the use of nudges to influence patient and clinician behavior in the context of clinical trials is still in its infancy. Further research is warranted to examine whether these strategies can be employed to influence enrollment and how they can be successfully implemented (e.g., feasible, acceptable, and sustainable) in both academic and community-based cancer practices. 

## 7. Conclusions

The underrepresentation of older adults in cancer clinical trials is undeniably a multifaceted problem that requires a multifaceted solution. There have been promising steps toward improving trial participation among older adults, but there are still significant gaps in knowledge that hinder older patients from receiving high-quality, individualized, evidence-based care. The recommendations presented in this review range from small changes that can be adopted by individuals and research teams to large-scale, systemic changes that are needed at the institutional and/or policy level. Regardless, multiple steps are needed on multiple fronts across a collaborative network of academic and community-based cancer practices in order to have a cumulative and palpable effect. An investment in academic and community clinical trial partnerships could help to further cancer research, and more importantly, ensure that older adults with cancer have equal access to new treatments and advances in care.

## Figures and Tables

**Table 1:** Clinical trial designs for geriatric oncology research.

Design	Description/Characteristics	Potential Objectives and Outcomes	Advantages	Limitations
Pragmatic Trial	Intervention typically performed in the context of standard care Patients recruited from a variety of practice settings, using broader, more inclusive eligibility criteria	Determine the effectiveness of an intervention in day-to-day practice	-More accessible -Less resource-intensive -Places minimal additional burden on participants -Outcomes relevant to those who will use trial results	-Non-adherence and loss to follow-up -Poor internal validity limits the ability to determine definitive effects
Randomized Controlled Trial	Subjects are randomly assigned to treatment arms To generate data on the older patient population: -Accrue only older adults, -Stratify enrollment into age groups Adaptive design: modifications are made as the study proceeds based on interim data analysis	Compare efficacy and tolerability of different treatments regimens	-Direct comparison of treatment regimens -Unbiased -Minimizes confounding -Generalizable to the overall population being studied	-Requires a large sample size -Costly and time-consuming -Logistically demanding -Slow accrual
Prospective Cohort Study	Assessment of treatments already approved by the FDA Cohort defined by host, tumor, or treatment factors Observational Hypothesis-driven	Understand decision-making, patterns of care	-Findings are generalizable	-Lack of randomization -Requires a large sample size -Logistically demanding
Extended Trial	Addition of a cohort of older patients to the superior treatment arm	Determine tolerability in older adults	Trial infrastructure already established Existing data on treatment efficacy will make accrual of older patients easier	-Lack of data on the inferior treatment arm in older adults
Embedded Study (Correlative or Ancillary Study)	Includes additional measures of interest in the infrastructure of a parent study	Describe a cohort or understand the impact of treatment using geriatric assessment measures	-Better understand the characteristics of the older patient population -Identification of predictors of functional decline	-In studies not specific to older adults, the sample size of older patients may be limited
N-of-1 (Single-Subject Trial)	Determines the optimal therapy for a single individual	Determine the optimal or best intervention for an individual patient using objective data-driven criteria	-Individualized medicine -Gain insights into comparative treatment effectiveness among a wide variety of patients	-Randomization of treatment order -Carryover effects -Wash-out periods -Blinding
